# Joint Estimation of 2D-DOA and Frequency Based on Space-Time Matrix and Conformal Array

**DOI:** 10.1155/2013/463828

**Published:** 2013-12-15

**Authors:** Liang-Tian Wan, Lu-Tao Liu, Wei-Jian Si, Zuo-Xi Tian

**Affiliations:** ^1^Department of Information and Communication Engineering, University of Harbin Engineering, Harbin 150001, China; ^2^Science and Technology on Underwater Test and Control Laboratory, Dalian 116013, China

## Abstract

Each element in the conformal array has a different pattern, which leads to the performance deterioration of the conventional high resolution direction-of-arrival (DOA) algorithms. In this paper, a joint frequency and two-dimension DOA (2D-DOA) estimation algorithm for conformal array are proposed. The delay correlation function is used to suppress noise. Both spatial and time sampling are utilized to construct the spatial-time matrix. The frequency and 2D-DOA estimation are accomplished based on parallel factor (PARAFAC) analysis without spectral peak searching and parameter pairing. The proposed algorithm needs only four guiding elements with precise positions to estimate frequency and 2D-DOA. Other instrumental elements can be arranged flexibly on the surface of the carrier. Simulation results demonstrate the effectiveness of the proposed algorithm.

## 1. Introduction

Conformal array is the array mounted on the surface of the conformal carrier [1]. The advantage of the conformal array includes the alleviation of array weight, reduction of aerodynamic drag, reduction of radar cross-section (RCS), and the cover of the whole range of azimuth [2]. Thus the conformal array is of great interest in a variety of applications such as radar, sonar, wireless communication, and seismology.

Conventional algorithms for direction-of-arrival (DOA) estimation, such as multiple signal classification (MUSIC) [3] and estimation of signal parameters via rotational invariance techniques (ESPRIT) [4], are not suitable for conformal array because of the varying curvature of the conformal carrier. Based on array interpolation [5, 6], the manifold separation technique was applied to arbitrary array structure [7]. The above algorithm possesses good performance when the pattern of element is isotropic directional. However, due to the effect of the curvature of conformal carrier, each element of the conformal array has different patterns [8]. Thus, the algorithm proposed in [7] could not be used for conformal array. Not all elements can receive signals because of the “shadow effect” caused by metallic carrier. The subarray divided technique and interpolation transformation were adopted for DOA estimation of conformal array. But the interpolation accuracy of virtual manifold transformation is not high [9, 10]. Recently, DOA estimation algorithms for conformal array have been proposed. Based on ESPRIT and specially designed array structure, a blind DOA estimation algorithm was presented [11]. The iterative ESPRIT algorithm was proposed for joint DOA and polarization parameter estimation [12]. Nevertheless, the two algorithms need parameter pairing and special array design.

By combining the techniques of temporal filtering and spatial beam forming, the joint DOA and delay were estimated [13]. Similarly, the proposed approach in [14] was a novel twist of parameter estimation and filtering processes, in which two 1D frequencies and one 1D spatial MUSIC were employed to estimate the DOAs and frequencies, respectively. Two approximate maximum likelihood (ML) algorithms were proposed in the spatially correlated noise scenario for joint frequency and DOA estimation. However, MUSIC and ML algorithms used in [13–15] suffer from tremendous computational complexity and are only suitable for linear array or planar array, which could not be used for conformal array. The research of joint frequency and DOA estimation is rare for the conformal array.

As a useful analysis tool of data arrays, parallel factor (PARAFAC) analysis model [16, 17] is a generalization of low-rank matrix decomposition to three-way arrays (TWAs) or multiway arrays (MWAs), which was introduced in array signal processing to estimate DOA [18]. PARAFAC has been first introduced as a data analysis tool in psychometrics, where it has been used in arithmetic complexity, exploratory data analysis, and other fields. The PARAFAC model was developed by Sidiropoulos et al. [19]. Most parameter estimation algorithms for conformal array focus on DOA estimation. The frequency estimation is not researched as far as we known. For 2D-DOA estimation of conformal array, the algorithms based on ESPRIT suffer from parameter pairing problem. The algorithms based on MUSIC suffer from tremendous computational complexity. In this paper, a novel joint frequency and 2D-DOA estimation algorithm for conformal array is proposed. The spatial domain sampling and time domain sampling are utilized to construct the spatial-time matrix. The delay correlation function is used to suppress noise. Based on PARAFAC, the joint frequency and 2D-DOA estimation are accomplished without parameter pairing. Unlike the special array design in [9–12], the proposed algorithm needs only four guiding elements, other instrumental elements of the array can be arranged flexibly.

The organization of this paper is structured as follows.  Section 2 introduces the structure of the conformal array and describes the snapshot data model.  Section 3 contains the core contributions of this paper, including the construction of spatial-time matrix and the PARAFAC model used for high accuracy frequency and 2D-DOA estimation.  Section 4 presents the simulation result.  Section 5 summarizes our conclusions.

## 2. The Structure of the Conformal Array and the Snapshot Data Model

**Figure 1:**
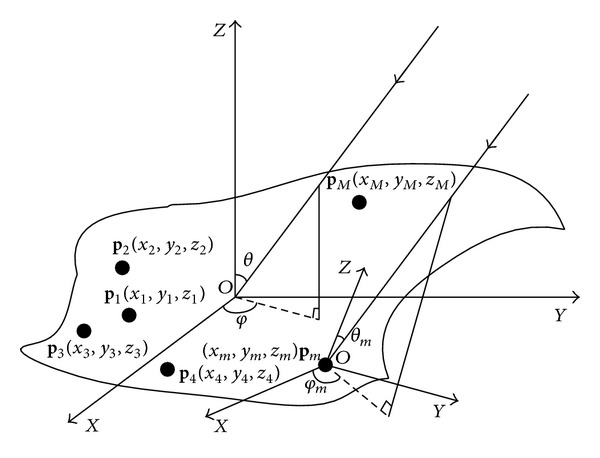
The structure of the conformal array.

A curve array antenna mounted on an arbitrary shape carrier is shown in Figure 1. Assuming that there are *M* elements on the surface of the carrier, the coordinate of each element can be represented as (*x*_*m*_, *y*_*m*_, *z*_*m*_),  *m* = 1,2,…, *M*. Four guiding elements **p**_1_, **p**_2_, **p**_3_, and **p**_4_ with precise positions are chosen, and **p**_1_ is assumed to be the reference element. **p**_*m*_ represents the position vector of the *m*th element on the surface of the carrier. ${\vec{e}}_{x}$, ${\vec{e}}_{y}$, and ${\vec{e}}_{z}$ represent the unit vector of *X*-axis, *Y*-axis, and *Z*-axis, respectively. **p**_*m*_ can be expressed as

(1) $$\begin{array}{c} p_{m}=x_{m}{\vec{e}}_{x}+y_{m}{\vec{e}}_{y}+z_{m}{\vec{e}}_{z}. \end{array}$$

Consider *K* narrow far field incident signals impinging on the conformal array, which is shown in Figure 1. The elevation and azimuth of *k*th incident signal are denoted as (*θ*_*k*_, *φ*_*k*_), *k* = 1,2,…, *K*. Thus, the general form of the array output is represented as

(2) $$\begin{array}{c} x_{m}\left(t\right)=\overset{K}{\underset{k=1}{\sum }}s_{k}\left(t\right)g_{m}\exp\left(-j\frac{2\pi f_{k}}{c}p_{m}•u_{k}\right), \end{array}$$

where *s*_*k*_(*t*) stands for the *k*th incident signal and *g*_*m*_ is the element pattern which is defined in the *m*th local coordinate. *f*_*k*_ is the frequency of the *k*th incident signal, *u*_*k*_ is the direction vector of the incident signal *s*_*k*_(*t*).

Consider

(3) $$\begin{array}{c} u_{k}=\sin\left(\theta_{k}\right)\cos\left(\varphi_{k}\right){\vec{e}}_{x}+\sin\left(\theta_{k}\right)\sin\left(\varphi_{k}\right){\vec{e}}_{y}+\cos\left(\theta_{k}\right){\vec{e}}_{z}. \end{array}$$

The time domain sampling is done for the signal of the array output. The number of the tapped delay line is *L*. Then the *l*th tapped delay of the signal output of the *m*th element can be represented as

(4) xm(t−lτ)=∑k=1Ksk(t)gmexp⁡[−jωk(τmk+lτ)]+nm(t−lτ), 0≤l≤L−1,

where *τ*_*mk*_ represents the time delay between the *m*th element **p**_*m*_ and reference element **p**_1_ when the *m*th element receives the *k*th incident signal, *τ* is the time interval of sampling, *n*_*m*_ is the additional white Gaussian noise (AWGN) with zero mean and variance *σ* ^2^, *ω*_*k*_ = 2*πf*_*k*_, and *τ*_*mk*_ = (**p**_*m*_•**u**_*k*_)/*c*.

The condition that the same incident signal impinges on the global coordinate and local coordinate is also shown in Figure 1. The coordinate which is constructed by the original point **p**_1_ is the global coordinate. The coordinate which is constructed by the original point **p**_*m*_ is the local coordinate. As shown in Figure 1, the elevation and azimuth of the incident signal in global coordinate and local coordinate are distinct. The pattern function *g*_*m*_(*θ*_*m*_, *φ*_*m*_) is defined in the local coordinate of each element. Thus, the pattern in the local coordinate of each element is different. Here the parameter component transformation from the global coordinate to the *m*th local coordinate can be accomplished by using the general Euler rotate method [8]. More details can be found in [20].

Due to the “shadow effect” caused by the metal, not all elements in the conformal array can receive the signal. Thus, the subarray divided technique [9, 10] is adopted and the whole array can be divided into several parts. The same parameter estimation mechanism is used for each part. For the sake of simplicity, it is assumed that all the elements can receive the signal.

## 3. Joint Frequency and 2D-DOA Estimation

### 3.1. The Construction of Spatial-Time Matrix

The delay correlation function between the *m*th element and *n*th element of the conformal array is constructed as follows:

(5) Rxmxn=E[xm(t)xn∗(t−τ)]=∑k=1KE[sk(t)sk∗(t−τ)]×exp⁡[−jωk(τmk−τnk−τ)]+E[nm(t)nn∗(t−τ)]=∑k=1KRsksk(τ)gmgnexp⁡[−jωk(τmk−τnk)]×exp⁡(jωkτ)+Rnmnn(τ), τ>0,

where **R**_*s*_*k*_*s*_*k*__(*τ*) = *E*[*s*_*m*_(*t*)*s*_n_*(*t* − *τ*)] represents the delay correlation function of *s*_*k*_(*t*), and **R**_*n*_*m*_*n*_*n*__(*τ*) = *E*[*n*_*m*_(*t*)*n*_n_*(*t* − *τ*)] represents the noise cross-correlation function between the *m*th element and *n*th element.

On condition that the incident signal is coming from narrow far field and the bandwidth of the incident signal is **B**, the delay correlation processing of the data that the elements receive takes a long period of time. Hence, **R**_*s*_*k*_*s*_*k*__(*τ*) = *E*[*s*_*m*_(*t*)*s*_n_*(*t* − *τ*)] ≠ 0. In the more general case, when *τ* ≪ 1/*B*, the Gaussian white noise is not correlated during the time interval *τ*. At the same time, the variety of the signal envelope can be neglected. Then we can suppress the noise without destroying the signal.

Consider

(6) Rnmnn(τ)=E[nm(t)nn∗(t−τ)]=σ2δ(τ)δ(m−n)=0,

where the delay correlation function is constructed between *x*_1_(*t* − *τ*) and the data that each element receives. Based on (5), we can obtain

(7) Rxmx1(τ)=E[xm(t)x1∗(t−τ)]=∑k=1KRsksk(τ)gmg1exp⁡[−jωk(τmk−τ1k)]×exp⁡(jωkτ), 1≤m≤M.

Similarly, the delay correlation functions are constructed between *x*_2_(*t* − *τ*), *x*_3_(*t* − *τ*), *x*_4_(*t* − *τ*), and *x*_1_(*t*) and the data that each element receives, respectively.

Consider

(8) Rxmx2(τ)=E[xm(t)x2∗(t−τ)]=∑k=1KRsksk(τ)gmg1exp⁡[−jωk(τmk−τ2k)]×g2g1exp⁡(jωkτ), 1≤m≤M,Rxmx3(τ)=E[xm(t)x3∗(t−τ)]=∑k=1KRsksk(τ)gmg1exp⁡[−jωk(τmk−τ3k)]×g3g1exp⁡(jωkτ), 1≤m≤M,Rxmx4(τ)=E[xm(t)x4∗(t−τ)]=∑k=1KRsksk(τ)gmg1exp⁡[−jωk(τmk−τ4k)]×g4g1exp⁡(jωkτ), 1≤m≤M,Rxmx1(0)=E[xm(t)x1∗(t)]=∑k=1KRsksk(0)gmg1exp⁡[−jωk(τmk−τ1k)]+σ2I, 1≤m≤M.

The spatial-time matrix is constructed as follows:

(9) $$\begin{array}{c} R_{S}\left(\tau \right)={\left[R_{s_{1}s_{1}}\left(\tau \right),R_{s_{2}s_{2}}\left(\tau \right),\ldots ,R_{s_{K}s_{K}}\left(\tau \right)\right]}^{T}, \end{array}$$

(10) $$\begin{array}{c} R_{f}\left(\tau \right)={\left[R_{x_{1}x_{1}}\left(\tau \right),R_{x_{2}x_{1}}\left(\tau \right),\ldots ,R_{x_{m}x_{1}}\left(\tau \right)\right]}^{T}, \end{array}$$

(11) $$\begin{array}{c} R_{1}\left(\tau \right)={\left[R_{x_{1}x_{2}}\left(\tau \right),R_{x_{2}x_{2}}\left(\tau \right),\ldots ,R_{x_{m}x_{2}}\left(\tau \right)\right]}^{T}, \end{array}$$

(12) $$\begin{array}{c} R_{2}\left(\tau \right)={\left[R_{x_{1}x_{3}}\left(\tau \right),R_{x_{2}x_{3}}\left(\tau \right),\ldots ,R_{x_{m}x_{3}}\left(\tau \right)\right]}^{T}, \end{array}$$

(13) $$\begin{array}{c} R_{3}\left(\tau \right)={\left[R_{x_{1}x_{4}}\left(\tau \right),R_{x_{2}x_{4}}\left(\tau \right),\ldots ,R_{x_{m}x_{4}}\left(\tau \right)\right]}^{T}, \end{array}$$

(14) $$\begin{array}{c} R\left(0\right)={\left[R_{x_{1}x_{1}}\left(0\right),R_{x_{2}x_{1}}\left(0\right),\ldots ,R_{x_{m}x_{1}}\left(0\right)\right]}^{T}, \end{array}$$

(15) $$\begin{array}{c} A=\left[a_{1}\left(\omega_{1}\right),a_{2}\left(\omega_{2}\right),\ldots ,a_{K}\left(\omega_{K}\right)\right], \end{array}$$

(16) ak(ωk)=[g1e(−j(ωk/c)p1•uk),g2e(−j(ωk/c)p2•uk) ,…,gMe(−j(ωk/c)pM•uk)]T.

The matrix **A** is the manifold matrix and **a** is the steering matrix. Then (9)–(14) can be written as

(17) $$\begin{array}{c} R_{f}\left(\tau \right)=A\Phi_{f}R_{S}\left(\tau \right), \end{array}$$

(18) $$\begin{array}{c} R_{1}\left(\tau \right)=A\Phi_{1}\Phi_{f}R_{S}\left(\tau \right), \end{array}$$

(19) $$\begin{array}{c} R_{2}\left(\tau \right)=A\Phi_{2}\Phi_{f}R_{S}\left(\tau \right), \end{array}$$

(20) $$\begin{array}{c} R_{3}\left(\tau \right)=A\Phi_{3}\Phi_{f}R_{S}\left(\tau \right), \end{array}$$

(21) $$\begin{array}{c} R\left(0\right)=AR_{S}\left(0\right), \end{array}$$

where

(22) $$\begin{array}{c} \Phi_{f}=\mathrm{diag}{\left[{{\text{e}}_{}}^{\left(j\omega_{1}\tau \right)},{{\text{e}}_{}}^{\left(j\omega_{2}\tau \right)},\ldots ,{{\text{e}}_{}}^{\left(j\omega_{K}\tau \right)}\right]}^{T}, \end{array}$$

(23) Φ1=diag⁡[g2g1e(j(ω1/c)(p2−p1)•u1),g2g1e(j(ω2/c)(p2−p1)•u2)    ,…,g2g1e(j(ωK/c)(p2−p1)•uK)]T,

(24) Φ2=diag⁡[g3g1e(j(ω1/c)(p3−p1)•u1),g3g1e(j(ω2/c)(p3−p1)•u2)    ,…,g3g1e(j(ωK/c)(p3−p1)•uK)]T,

(25) Φ3=diag⁡[g4g1e(j(ω1/c)(p4−p1)•u1),g4g1e(j(ω2/c)(p4−p1)•u2)    ,…,g4g1e(j(ωK/c)(p4−p1)•uK)]T,

(26) $$\begin{array}{c} \eta_{fk}=\omega_{k}\tau =2\pi f_{k}\tau , \end{array}$$

(27) $$\begin{array}{c} \eta_{1k}=\frac{\omega_{k}}{c}\left(p_{2}-p_{1}\right)•u_{i}, \end{array}$$

(28) $$\begin{array}{c} \eta_{2k}=\frac{\omega_{k}}{c}\left(p_{3}-p_{1}\right)•u_{i}, \end{array}$$

(29) $$\begin{array}{c} \eta_{3k}=\frac{\omega_{k}}{c}\left(p_{4}-p_{1}\right)•u_{i}. \end{array}$$

The incident signal is narrowband; thus, **R**_*s*_*k*_*s*_*k*__(*nτ*) = **R**_*s*_*k*_*s*_*k*__((*n* − 1)*τ*). Equation (21) can be written in another form as

(30) $$\begin{array}{c} R\left(\tau \right)=AR_{S}\left(\tau \right). \end{array}$$

Equations (17)–(21) possess the analogous form. **R**_*f*_(*τ*), **R**_1_(*τ*), **R**_2_(*τ*), **R**_3_(*τ*), and **R**(0) are sampled at a time delay *τ*_*s*_ for *N* (*N* ≥ *K*) times, *τ*_*s*_ = *T*_*s*_, 2*T*_*s*_,…, *NT*_*s*_, and *T*_*s*_ is the time interval of sampling which should be less than the reciprocal of the highest frequency. Thus, the aliasing effect would not happen. The pseudosnapshot data matrix can be constructed by using (17)–(21) as follows:

(31) $$\begin{array}{c} R_{f}=\left[R\left(T_{s}\right),R\left(2T_{s}\right),\ldots ,R\left(NT_{s}\right)\right],\\ R_{1}=\left[R_{1}\left(T_{s}\right),R_{1}\left(2T_{s}\right),\ldots ,R_{1}\left(NT_{s}\right)\right],\\ R_{2}=\left[R_{2}\left(T_{s}\right),R_{2}\left(2T_{s}\right),\ldots ,R_{2}\left(NT_{s}\right)\right],\\ R_{3}=\left[R_{3}\left(T_{s}\right),R_{3}\left(2T_{s}\right),\ldots ,R_{3}\left(NT_{s}\right)\right],\\ R=\left[R_{f}\left(T_{s}\right),R_{f}\left(2T_{s}\right),\ldots ,R_{f}\left(NT_{s}\right)\right]. \end{array}$$

Equation (31) can be written as

(32) $$\begin{array}{c} R_{f}=A\Phi_{f}R_{S},\\ R_{1}=A\Phi_{1}\Phi_{f}R_{S},\\ R_{2}=A\Phi_{2}\Phi_{f}R_{S},\\ R_{3}=A\Phi_{3}\Phi_{f}R_{S},\\ R=AR_{S}, \end{array}$$

where

(33) $$\begin{array}{c} R_{S}=\left[R_{S}\left(T_{s}\right),R_{S}\left(2T_{s}\right),\ldots ,R_{S}\left(NT_{s}\right)\right]. \end{array}$$

### 3.2. PARAFAC

The element of a tensor $\underline{X}\in {𝒞}^{C\times D\times E}$ is *x*_*i*,*j*,*k*_ and its *P*-component PARAFAC decomposition is given by

(34) $$\begin{array}{c} x_{c,d,e}=\overset{M}{\underset{p=1}{\sum }}{\bar{s}}_{c,p}{\bar{t}}_{d,p}{\bar{u}}_{e,p}, \end{array}$$

where *c* = 1,…, *C*, *d* = 1,…, *D*, and *e* = 1,…, *E*. ${\bar{s}}_{c,p}$, ${\bar{t}}_{d,p}$, and ${\bar{u}}_{e,p}$ stand for the elements of $\bar{S}\in {𝒞}^{C\times P}$, $\bar{T}\in {𝒞}^{D\times P}$, and $\bar{U}\in {𝒞}^{E\times P}$, respectively. The typical elements of the **X**_*c*_ ∈ *𝒞* ^*D*×*E*^, **X**_*d*_ ∈ *𝒞* ^*E*×*C*^, and **X**_*e*_ ∈ *𝒞* ^*C*×*D*^are *X*_*c*_(*d*, *e*) : = *X*_*d*_(*c*, *e*) : = *X*_*e*_(*c*, *d*) : = *x*_*c*,*d*,*e*_, respectively. The three way array (TWA) $\underline{X}$ is “sliced” in three different directions:

(35) $$\begin{array}{c} X_{c}=\bar{T}\Lambda_{c}\left(\bar{S}\right){\bar{U}}^{T},\;c=1,\ldots ,C,\\ X_{d}=\bar{U}\Lambda_{d}\left(\bar{T}\right){\bar{S}}^{T},\;d=1,\ldots ,D,\\ X_{e}=\bar{S}\Lambda_{e}\left(\bar{U}\right){\bar{T}}^{T},\;e=1,\ldots ,E. \end{array}$$

For a given matrix $\bar{S}$, $\Lambda_{c}\left(\bar{S}\right)$ means a diagonal matrix with the diagonals given by the *c*th row of the matrix. The symbol “⊙” is used to denote the Khatri-Rao (KR) product. As an example, the KR product of two matrices $\bar{S}\in {𝒞}^{C\times P}$ and $\bar{T}\in {𝒞}^{D\times P}$ of an identical number of columns is given by

(36) $$\begin{array}{c} \bar{S}⊙\bar{T}=\left[{\bar{s}}_{1}⊗{\bar{t}}_{1},\ldots ,{\bar{s}}_{P}⊗{\bar{t}}_{P}\right]\in {𝒞}^{CD\times P}, \end{array}$$

where “⊗” represents the Kronecker product. Then the definition of Kronecker product of two vectors $\bar{s}\in {𝒞}^{C}$ and $\bar{t}\in {𝒞}^{D}$ is given by

(37) $$\begin{array}{c} \bar{s}⊗\bar{t}=\left[\begin{array}{c} {\bar{s}}_{1}\bar{t}\\ {\bar{s}}_{2}\bar{t}\\ \vdots \\ {\bar{s}}_{C}\bar{t} \end{array}\right]. \end{array}$$

Based on the definition of KR product, **X** ^*CD*×*E*^ can be expressed as

(38) $$\begin{array}{c} X^{CD\times E}=\left|\begin{array}{c} X_{c=1}\\ X_{c=2}\\ \vdots \\ X_{c=C} \end{array}\right|=\left|\begin{array}{c} {\bar{T}\Lambda }_{1}\left(\bar{S}\right)\\ {\bar{T}\Lambda }_{2}\left(\bar{S}\right)\\ \vdots \\ \bar{T}\Lambda_{C}\left(\bar{S}\right) \end{array}\right|{\bar{U}}^{T}=\left(\bar{S}⊙\bar{T}\right){\bar{U}}^{T}. \end{array}$$

Similarly, the matrix **X** ^*DE*×*C*^ and **X** ^*EC*×*D*^ can be expressed as

(39) $$\begin{array}{c} X^{DE\times C}=\left(\bar{T}⊙\bar{U}\right){\bar{S}}^{T},\\ X^{EC\times D}=\left(\bar{U}⊙\bar{S}\right){\bar{T}}^{T}. \end{array}$$

**Definition 1 (Kruskal rank [18])**

The Kruskal rank, or k-rank of a given matrix $\bar{S}\in {𝒞}^{C\times P}$ denoted by krank ($\bar{S}$) is said to be equal to *r* when every collection of *r* columns of ($\bar{S}$) is linearly independent but there exists a collection of *r* + 1 linearly dependent columns. However, the rank of $\bar{S}$ is said to be the maximal number of linearly independent columns. The definition of $\mathrm{rank}\left(\bar{S}\right)$ is more relaxed than that of $\text{krank}\left(\bar{S}\right)$; that is, $\text{krank}\left(\bar{S}\right)\leq \mathrm{rank}\left(\bar{S}\right)$.

**Theorem 2 (Kruskal theorem [19])**

Given a PARAFAC model $\underline{X}\in {𝒞}^{C\times D\times E}$; then the decomposition of this PARAFAC model is unique to permutation and scaling when

(40) $$\begin{array}{c} k_{\bar{S}}+k_{\bar{T}}+k_{\bar{U}}\geq 2P+2. \end{array}$$

The matrix **X** is constructed by $\hat{S}$, $\hat{T}$, and $\hat{U}$ as

(41) $$\begin{array}{c} \hat{S}=\bar{S}\Pi \Delta_{1},\;\;\hat{T}=\bar{T}\Pi \Delta_{2},\;\;\hat{U}=\bar{U}\Pi \Delta_{3}, \end{array}$$

where Π stands for the permutation matrix. Δ_1_, Δ_2_, and Δ_3_ are diagonal scaling matrices satisfying

(42) $$\begin{array}{c} \Delta_{1}\Delta_{2}\Delta_{3}=I, \end{array}$$

where **I** is a unit matrix.

The *M* × *M* × 5 TWA of the conformal array is constructed based on PARAFAC model.

(43) $$\begin{array}{c} \hat{R}=\left|\begin{array}{c} R\left(:,:,1\right)\\ R\left(:,:,2\right)\\ R\left(:,:,3\right)\\ R\left(:,:,4\right)\\ R\left(:,:,5\right) \end{array}\right|=\left|\begin{array}{c} R_{f}\\ R_{1}\\ R_{2}\\ R_{3}\\ R \end{array}\right|=\left|\begin{array}{c} A\Phi_{f}R_{S}\\ A\Phi_{1}\Phi_{f}R_{S}\\ A\Phi_{2}\Phi_{f}R_{S}\\ A\Phi_{3}\Phi_{f}R_{S}\\ AR_{S} \end{array}\right|+\hat{Q}, \end{array}$$

where $\hat{Q}$ represents the observed noise in practice. In order to make the decomposition of the PARAFAC unique, the following assumption must be satisfied:

(44) $$\begin{array}{c} a_{i}\left(\omega_{i}\right)\neq a_{j}\left(\omega_{j}\right). \end{array}$$

In other words, for two incident signals *s*_*i*_(*t*) and *s*_*j*_(*t*) with different DOAs, the following condition must be satisfied

(45) $$\begin{array}{c} g_{oe}{\text{e}}^{-j2\pi f_{i}(\left(p_{oe}•u_{i}\right)/c)}\neq g_{oe}{\text{e}}^{-j2\pi f_{j}(\left(p_{oe}•u_{j}\right)/c)}. \end{array}$$

The k-rank of matrix **D**, **A**, and **R**_*S*_ are *k*_*D*_ = min⁡(5, *K*), *k*_*A*_ = *K*, and *k*_*R*_*S*__ = *K*, respectively. Thus, when *K* ≥ 2, the k-rank decomposition of the PARAFAC is unique.

Based on the definition of KR product, (43) can be written in three forms as follows:

(46) $$\begin{array}{c} R=\left(D⊙A\right)R_{S}+\hat{Q}, \end{array}$$

(47) $$\begin{array}{c} R_{X}=\left(R_{S}^{T}⊙D\right)A^{T}+{\hat{Q}}_{X}, \end{array}$$

(48) $$\begin{array}{c} R_{Y}=\left(A⊙R_{S}^{T}\right)D^{T}+{\hat{Q}}_{Y}, \end{array}$$

where

(49) $$\begin{array}{c} D=\left|\begin{array}{c} \Lambda^{-1}\left(\Phi_{f}\right)\\ \Lambda^{-1}\left(\Phi_{1}\Phi_{f}\right)\\ \Lambda^{-1}\left(\Phi_{2}\Phi_{f}\right)\\ \Lambda^{-1}\left(\Phi_{3}\Phi_{f}\right)\\ \Lambda^{-1}\left(1\right) \end{array}\right|, \end{array}$$

where Λ^−1^(Φ_*f*_) means the row vector constructed by the diagonal elements of the diagonal matrix Φ_*f*_.

The matrices **D**, **A**, and **R**_*S*_ can be solved by the trilinear alternating least squares (TALS) regression algorithm. Due to the existence of the observed noise, (46) could be transformed as a least square problem as follows:

(50) $$\begin{array}{c} \underset{D,A,R_{S}}{\min}{||\hat{R}-\left(D⊙A\right)R_{S}||}_{F}^{2}. \end{array}$$

Then the least square estimator of **R**_*S*_ can be expressed as

(51) $$\begin{array}{c} R_{S}=\arg\underset{R_{S}}{\min}{||\hat{R}-\left(D⊙A\right)R_{S}||}_{F}^{2}. \end{array}$$

Similarly, the solutions of **D** and **A** can be expressed as

(52) $$\begin{array}{c} A^{T}={\left(R_{S}^{T}⊙D\right)}^{†}R_{X}\\ D^{T}={\left(A⊙R_{S}^{T}\right)}^{†}R_{Y}. \end{array}$$

We can update the matrices **D**, **A**, and **R**_*S*_ alternately until the algorithm converges; the estimators of the three matrices can be acquired. In the following experiments, the COMFAC algorithm (the TALS regression algorithm is realized in practice) is used to fit the *m* × *m* × 5 TWA. The initialization and fitting of the COMFAC is done in compressed space. The Tucker 3 three-way model is used in data compression [21].

Now the ALS algorithm steps can be summarized as follows.

1. Initialize **A** ^(0)^ ∈ *𝒞* ^*M*×*K*^ and **D** ^(0)^ ∈ *𝒞* ^5×*K*^.
2. Initialize *ε* > 0 and *k* = 0.
3. If ||*ρ* ^(*k*+1)^ − *ρ* ^(*k*)^||/*ρ* ^(*k*)^ > *ε*, calculate matrices **A**, **R**_*S*_, and **D** by (51)-(52). Update just one matrix at each time; then *k* → *k* + 1.
4. Else ||*ρ* ^(*k*+1)^ − *ρ* ^(*k*)^||/*ρ* ^(*k*)^ < *ε*; the iteration is terminated.

In this paper, *ρ* stands for **A**, **R**_*S*_, and **D**, respectively.

### 3.3. The Joint Parameter Estimation

The matrix **D** can be estimated by TALS regression algorithm; then the estimator of *ω*_*fk*_ is given by

(53) $$\begin{array}{c} \omega_{ki}=\text{angle}\left|\frac{D_{1k}}{D_{5k}}\right|. \end{array}$$

Because *r*_1_ and *r*_2_ are real numbers, **D**_2*k*_/**D**_1*k*_ is squared to avoid the ambiguity caused by the positive and negative values of *r*_1_ and *r*_2_. Then

(54) ω1k=−12angle([g2(θk,φk)g1(θk,φk)exp⁡(−jω1k)]2)=−12angle(exp⁡(−j2ω1k))=−12angle([D2kD1k]2).

Similarly

(55) $$\begin{array}{c} \omega_{2k}=-\frac{1}{2}\text{angle}\left({\left[\frac{D_{3k}}{D_{1k}}\right]}^{2}\right) \end{array}$$

(56) $$\begin{array}{c} \omega_{3k}=-\frac{1}{2}\text{angle}\left({\left[\frac{D_{4k}}{D_{1k}}\right]}^{2}\right). \end{array}$$

Taking (26) into (53), the frequency *f*_*k*_ of the *k*th incident signal is expressed as

(57) $$\begin{array}{c} f_{k}=\frac{1}{2\pi \tau }\text{angle}\left|\frac{D_{1k}}{D_{5k}}\right|. \end{array}$$

Assuming that *γ*_1*k*_ = sin(*θ*_*k*_)cos⁡(*φ*_*k*_), *γ*_2*k*_ = sin(*θ*_*k*_)sin(*φ*_*k*_), and *γ*_3*k*_ = cos⁡(*θ*_*k*_), and solving (27), (28), (29), (55), (56), and (57) simultaneously, we can obtain the equation as follows:

(58) $$\begin{array}{c} \frac{c}{2\pi f_{k}}\left[\begin{array}{c} \frac{\omega_{1k}}{d_{1}}\\ \frac{\omega_{2k}}{d_{2}}\\ \frac{\omega_{3k}}{d_{3}} \end{array}\right]=\left[\begin{array}{ccc} x_{2}-x_{1} & y_{2}-y_{1} & z_{2}-z_{1}\\ x_{3}-x_{1} & y_{3}-y_{1} & z_{3}-z_{1}\\ x_{4}-x_{1} & y_{4}-y_{1} & y_{4}-y_{1} \end{array}\right]\left[\begin{array}{c} \gamma_{1k}\\ \gamma_{2k}\\ \gamma_{3k} \end{array}\right]. \end{array}$$

The solution of the equation is represented as

(59) $$\begin{array}{c} \left[\begin{array}{c} \gamma_{1k}\\ \gamma_{2k}\\ \gamma_{3k} \end{array}\right]=\frac{c}{2\pi f_{i}}{\left[\begin{array}{ccc} x_{2}-x_{1} & y_{2}-y_{1} & z_{2}-z_{1}\\ x_{3}-x_{1} & y_{3}-y_{1} & z_{3}-z_{1}\\ x_{4}-x_{1} & y_{4}-y_{1} & y_{4}-y_{1} \end{array}\right]}^{-1}\left[\begin{array}{c} \frac{\omega_{1k}}{d_{1}}\\ \frac{\omega_{2k}}{d_{2}}\\ \frac{\omega_{3k}}{d_{3}} \end{array}\right]. \end{array}$$

There are two approaches to solve *θ*_*k*_ and *φ*_*k*_. The first approach is

(60) $$\begin{array}{c} \theta_{k}=\text{arccos}\gamma_{3k},\\ \varphi_{k}=\arcsin\left(\frac{\gamma_{1k}}{\cos\left(\theta_{k}\right)}\right)\;\text{or}\;\;\varphi_{k}=\arcsin\left(\frac{\gamma_{2k}}{\sin\left(\theta_{k}\right)}\right). \end{array}$$

The second approach is

(61) $$\begin{array}{c} \varphi_{k}=\arctan\left(\frac{\gamma_{2k}}{\gamma_{1k}}\right),\\ \theta_{k}=\text{arccos}\left(\frac{\gamma_{1k}}{\cos\left(\varphi_{k}\right)}\right)\;\text{or}\;\;\theta_{k}=\arcsin\left(\frac{\gamma_{2k}}{\sin\left(\varphi_{k}\right)}\right). \end{array}$$

In this paper the first approach is employed.

In order to ensure the uniqueness of the estimated parameters, the following condition must be satisfied:

(62) $$\begin{array}{c} ||p_{2}-p_{1}||\leq \frac{c}{\left(2\max\left(f_{i}\right)\right)}=\frac{\min\left(\lambda_{k}\right)}{2},\\ ||p_{3}-p_{1}||\leq \frac{c}{\left(2\max\left(f_{i}\right)\right)}=\frac{\min\left(\lambda_{k}\right)}{2},\\ ||p_{4}-p_{1}||\leq \frac{c}{\left(2\max\left(f_{i}\right)\right)}=\frac{\min\left(\lambda_{k}\right)}{2}. \end{array}$$

According to Theorem 2, the matrices **D**, **A**, and **R**_*S*_ have the same permutation matrix, that is, the *k*th column of matrix **D** corresponds to the *k*th column of the manifold matrix **A**.

Based on the PARAFAC model and TALS regression algorithm, the joint frequency and 2D-DOA estimation can be accomplished. The algorithm steps are summarized as follows:

1. choose four guiding elements and obtain the accurate positions of the elements;
2. calculate the delay correlation function between the four elements mentioned above and the whole elements of the conformal array;
3. calculate the matrices **R**_*f*_(*τ*), **R**_1_(*τ*), **R**_2_(*τ*), **R**_3_(*τ*), and **R**(0), respectively according to (5);
4. calculate the pseudosnapshot data matrix based on (17)–(21). **R**_*f*_(*τ*), **R**_1_(*τ*), **R**_2_(*τ*), **R**_3_(*τ*), and **R**(0) are sampled at time delay *τ*_*s*_, respectively;
5. construct the PARAFAC model based on (43);
6. estimate the matrix **D** using TALS regression algorithm;
7. obtain *ω*_*fk*_, *ω*_1*k*_, *ω*_2*k*_, and *ω*_3*k*_ by using the estimator of the matrix **D** and calculating (53)–(56);
8. obtain the joint frequency and 2D-DOA estimation by calculate (57)–(59) or (57), (60), and (61).

## 4. Simulation Results

This section focuses on several sets of simulation results to demonstrate the performance of the proposed algorithm. In all simulation examples, 200 Monte Carlo trials are considered, and the root mean square error (RMSE) is used as our performance measure, which is defined as

(63) $$\begin{array}{c} \text{RMSE}=\sqrt{\frac{1}{200}\overset{200}{\underset{\eta =1}{\sum }}\left[{\left({\hat{\theta }}_{k,\eta }-\theta \right)}^{2}+{\left({\hat{\varphi }}_{k,\eta }-\varphi \right)}^{2}\right]}, \end{array}$$

where ${\hat{\theta }}_{i,t}$ and ${\hat{\varphi }}_{i,t}$ are the elevation and azimuth estimators of the *k*th incident signal in the *η*th trial.

The successful rate is defined as the proportion of the number of the successful experiments to the total number of the experiments. For frequency estimation, a successful experiment is defined as the experiment with estimation error of less than 2 MHz; for DOA estimation, a successful experiment is defined as the experiment with estimation error of less than 2 degree.

**Figure 2:**
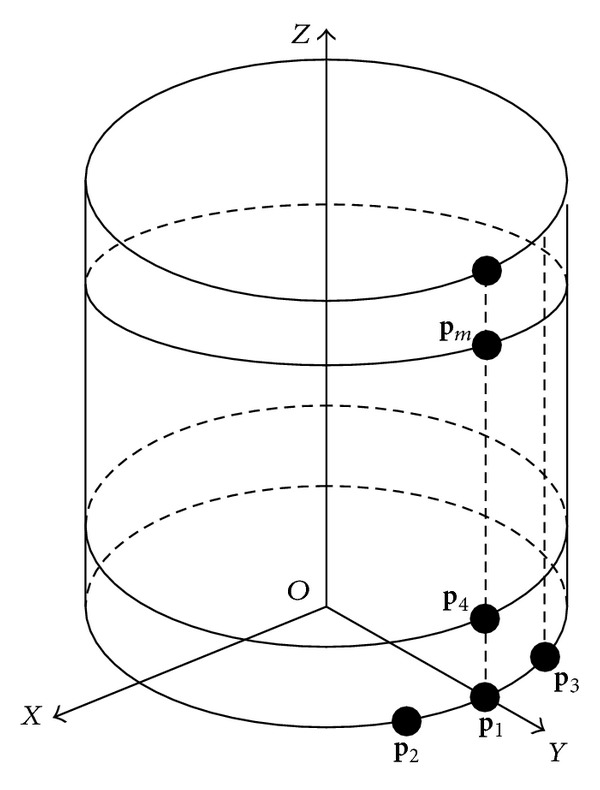
The structure of the cylindrical conformal array.

In order to simplify the transformation from the global coordinate to the local coordinate, only the cylinder with single curvature is considered in the simulations. The array structure is shown in Figure 2 is only a subarray, which only covers 120°. Then three subarrays can estimate the whole space. The highest frequency of the incident signal is *f*_max⁡_ = 2 GHz, *λ*_min⁡_ = *c*/*f*_max⁡_. The radius of the cylinder is 5*λ*_min⁡_, the adjacent element spacing in the same intersecting surface of the cylinder is *λ*_min⁡_/4, and the adjacent intersecting surface spacing is *λ*_min⁡_/4, *λ*_min⁡_ stands for the shortest wavelength of the incident signal. The position of the reference element is *p*_1_ = (0,5*λ*, 0).

The element pattern is defined in the local coordinate of each element; thus, the transformation from the global coordinate to the local coordinate of elevation *θ*_*k*_ and azimuth *φ*_*k*_ of the incident signal must be accomplished. For the cylindrical conformal array, the corresponding Euler rotation angles are

(64) $$\begin{array}{c} D=-\Theta ,\;\;E=-\pi /2,\;\;F=0, \end{array}$$

respectively. *D* represents the rotation angle based on the right-hand rule in the first rotation and the *Z*-axis is the rotation axis; *E* represents the rotation angle in the second rotation and the *Y*-axis is the rotation axis; and *F* represents the rotation angle in the third rotation and the *X*-axis is the rotation axis. Θ represents the angle between the position vector of the element and the positive direction of *X*-axis.

The unit vector of the *k*th incident signal (*θ*_*k*_, *φ*_*k*_) in the global coordinate can be expressed as

(65) $$\begin{array}{c} x=\sin\left(\theta_{k}\right)\cos\left(\varphi_{k}\right),\;\;y=\sin\left(\theta_{k}\right)\sin\left(\varphi_{k}\right),\\ z=\cos\left(\theta_{k}\right). \end{array}$$

Based on the Euler rotation matrix, the unit vector in global coordinate can be transformed into the local coordinate of the *m*th element.

Consider

(66) $$\begin{array}{c} {\left[\begin{array}{ccc} x_{m} & y_{m} & z_{m} \end{array}\right]}^{T}=R\left(D_{m},E_{m},F_{m}\right){\left[\begin{array}{ccc} x & y & z \end{array}\right]}^{T}, \end{array}$$

where

(67) R(Dm,Em,Fm)=[cos⁡FsinF0−sinFcos⁡F0001]×[cos⁡E0−sinE010sinE0cos⁡E]×[cos⁡DsinD0−sinDcos⁡D0001].

Then the elevation *θ* and azimuth *φ* of the *k*th incident signal in the local coordinate of the *m*th element can be represented as

(68) $$\begin{array}{c} \varphi_{mk}=\arctan\left(y_{m}/x_{m}\right),\;\;\theta_{mk}=\text{arccos}\left(z_{m}\right). \end{array}$$

Thus, the element pattern transformation from the global coordinate to the local coordinate is completed.

**Figure 3:**
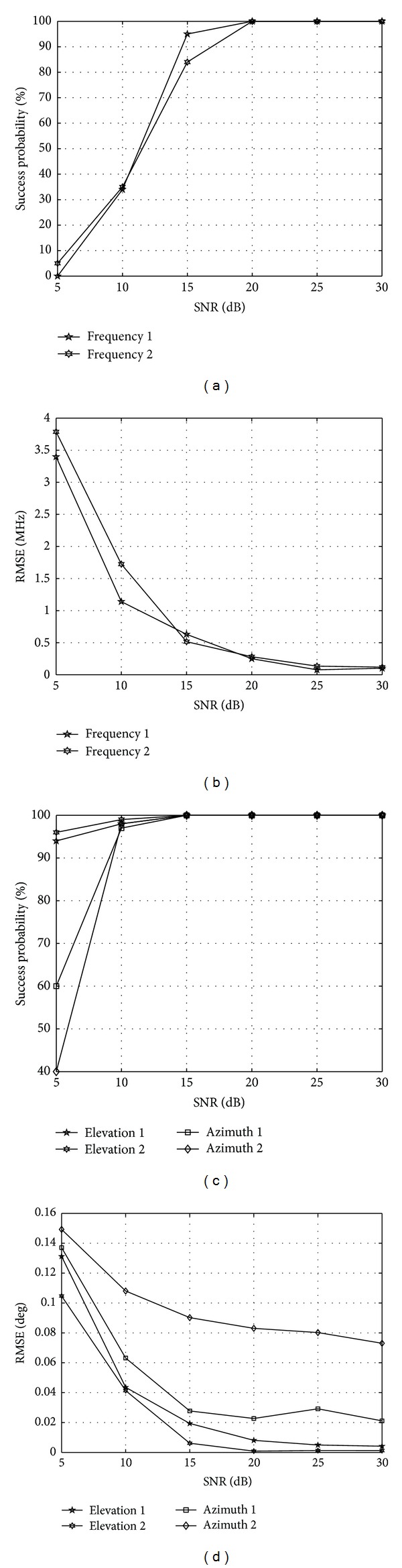
The RMSE and successful probability with different SNR. (a) The successful probability of frequencies, (b) the RMSE of frequency, (c) the successful probability of angles, and (d) the RMSE of angles.

First, the curves of RMSE of frequency and angle and the successful probability of angle in different SNR are plotted in Figure 3 using the proposed algorithm. The number of snapshots is 200, the number of pseudosnapshots is 100. SNR ∈ [5,30], and the noise is AWGN which is independent of incident signals. The sampling frequency is *f*_*s*_ = 1/*T*_*s*_ = 5 GHz. The elevation *θ*, azimuth *φ*, and frequency of these two narrow incident signals are (100°, 60°, 1 GHz) and (95°, 50°, 2 GHz), respectively, which are independent of each other. The number of elements are 10, including 4 guiding elements and other 6 instrumental elements. The element pattern used in simulation is the lowest order circular patch model [8, 22]

(69) gθ(θ,φ)={J2(πdλsinθ)−J0(πdλsinθ)}×(cos⁡φ−jsinφ), 0≤θ≤π2,gφ(θ,φ)={J2(πdλsinθ)+J0(πdλsinθ)}×cos⁡θ(sinφ−jcos⁡φ), 0≤θ≤π2,gθ(θ,φ)=gφ(θ,φ)=0, otherwise,

where *J*_0_ and *J*_2_ are the zeroth- and second-order Bessel functions of the first kind. The element pattern transformation is achieved with the method proposed above.

**Figure 4:**
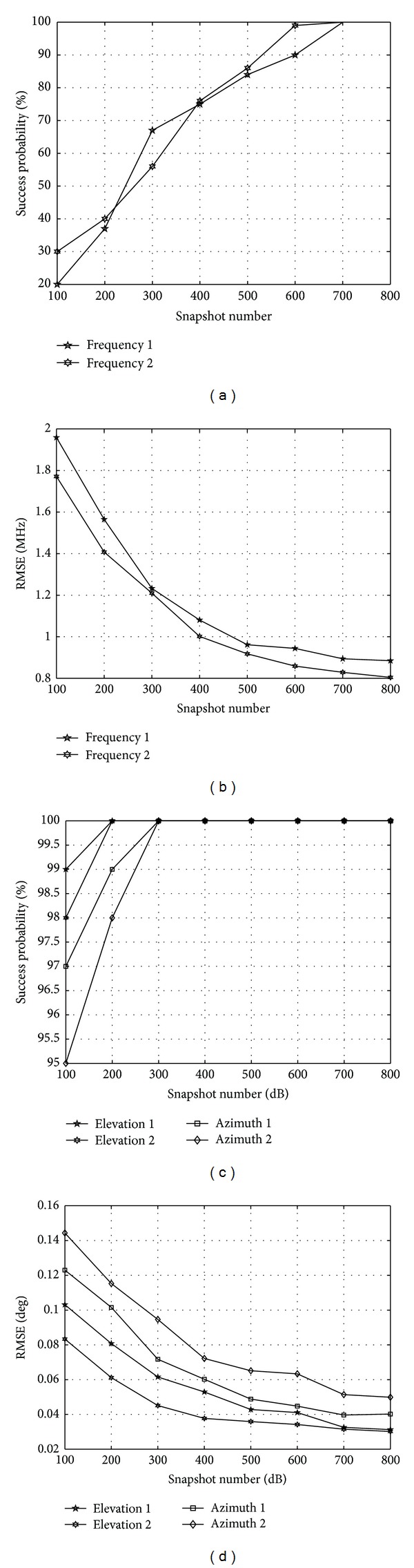
The RMSE and successful probability with different snapshot number. (a) The successful probability of frequencies, (b) the RMSE of frequency, (c) the successful probability of angles, and (d) the RMSE of angles.

Figure 3 shows that the RMSE of frequency and angle decreases while the SNR increases; meanwhile, the success probability of frequency and angle increase as the SNR increases. The estimated performance of the two frequencies are very close which can be seen in Figures 3(a) and 3(b). As shown in Figure 3(a), the successful probability is larger than 80%, when the SNR is larger than 15 dB. The RMSE of frequency is less than 2 MHz, when SNR is larger than 10 dB as shown in Figure 3(b). In Figure 3(c) the successful probability of azimuths is slightly lower than that of the elevations. The success probability of the angle is almost 100% when the SNR reaches 15 dB.  Figure 3(d) shows that the RMSE is smaller than 0.16° when SNR is larger than 5 dB. The RMSE of azimuth is larger than that of elevation. The estimation results of frequency and elevation are applied to estimate azimuth. The phenomenon in Figure 3(d) can be interpreted as the existence of the estimated error of frequency and elevation. Next, the RMSE of frequency and angle as well as the success probability of angle in different snapshot number are depicted in Figure 4. SNR = 10 dB; other simulation conditions are identical with the previous experiment. In Figure 4, the RMSE of frequency and angle decreases as the snapshot number increases, and the success probability of frequency and angle rises as the snapshot number increases. As shown in Figure 4(a), the successful probability is larger than 80%, when the number of snapshot is larger than 500.  Figure 4(b) shows that the RMSE of frequency 1 is slightly larger than that of frequency 2. When the snapshot number is larger than 500, the RMSE drops below 1 MHz, meaning that the proposed algorithm achieved three orders of magnitude reduction. Figures 4(c) and 4(d) show that the success probability of azimuth is lower than that of the elevation, and the RMSE of azimuth are larger than that of elevation. The reason why this happens is because the estimation error of frequency and elevation affects the estimation performance of azimuth.

## 5. Conclusion

Due to the varying curvature of the surface of the conformal carrier, the pattern of each element of the conformal array is different. Thus, the conventional algorithms could not be used for conformal array. In this paper, a novel joint frequency and 2D-DOA estimation algorithm with high accuracy are proposed. Both spatial and time sampling are utilized to construct the spatial-time matrix. The delay correlation function is used to suppress noise. The PARAFAC model is used for parameter estimation without parameter pairing. Only four elements are needed, and the positions of these elements should be known accurately. Other instrumental elements can be flexibly arranged on the surface of the conformal carrier. The algorithm proposed in this paper can be extended to estimate 2D-DOA straightly with little modification. The simulation results verify the effectiveness of the proposed algorithm. It can be expected that the proposed algorithm would have an application prospect in the parameter estimation of conformal array. In the future work, we will focus on the application of the proposed algorithm [23–26].
